# Exposure to Polyfluoroalkyl Chemicals and Attention Deficit/Hyperactivity Disorder in U.S. Children 12–15 Years of Age

**DOI:** 10.1289/ehp.1001898

**Published:** 2010-06-15

**Authors:** Kate Hoffman, Thomas F. Webster, Marc G. Weisskopf, Janice Weinberg, Verónica M. Vieira

**Affiliations:** 1 Department of Environmental Health, Boston University School of Public Health, Boston, Massachusetts, USA;; 2 Department of Environmental Health, Environmental and Occupational Medicine and Epidemiology, Harvard School of Public Health, Boston, Massachusetts, USA;; 3 Department of Biostatistics, Boston University School of Public Health, Boston, Massachusetts, USA

**Keywords:** attention deficit/hyperactivity disorder (ADHD), National Health and Nutrition Examination Survey (NHANES), perfluorohexane sulfonic acid (PFHxS), perfluorononanoic acid (PFNA), perfluorooctane sulfonic acid (PFOS), perfluorooctanoic acid (PFOA), polyfluoroalkyl chemicals (PFCs)

## Abstract

**Background:**

Polyfluoroalkyl chemicals (PFCs) have been widely used in consumer products. Exposures in the United States and in world populations are widespread. PFC exposures have been linked to various health impacts, and data in animals suggest that PFCs may be potential developmental neurotoxicants.

**Objectives:**

We evaluated the associations between exposures to four PFCs and parental report of diagnosis of attention deficit/hyperactivity disorder (ADHD).

**Methods:**

Data were obtained from the National Health and Nutrition Examination Survey (NHANES) 1999–2000 and 2003–2004 for children 12–15 years of age. Parental report of a previous diagnosis by a doctor or health care professional of ADHD in the child was the primary outcome measure. Perfluorooctane sulfonic acid (PFOS), perfluorooctanoic acid (PFOA), perfluorononanoic acid (PFNA), and perfluorohexane sulfonic acid (PFHxS) levels were measured in serum samples from each child.

**Results:**

Parents reported that 48 of 571 children included in the analysis had been diagnosed with ADHD. The adjusted odds ratio (OR) for parentally reported ADHD in association with a 1-μg/L increase in serum PFOS (modeled as a continuous predictor) was 1.03 [95% confidence interval (CI), 1.01–1.05]. Adjusted ORs for 1-μg/L increases in PFOA and PFHxS were also statistically significant (PFOA: OR = 1.12; 95% CI, 1.01–1.23; PFHxS: OR = 1.06; 95% CI, 1.02–1.11), and we observed a nonsignificant positive association with PFNA (OR = 1.32; 95% CI, 0.86–2.02).

**Conclusions:**

Our results, using cross-sectional data, are consistent with increased odds of ADHD in children with higher serum PFC levels. Given the extremely prevalent exposure to PFCs, follow-up of these data with cohort studies is needed.

Polyfluoroalkyl chemicals (PFCs) are a class of highly stable man-made compounds. Composed of a variable-length fluorinated carbon backbone and a carboxylate or sulfonate functional group, PFCs have both hydrophobic and oleophobic portions that enable products to repel both oil and water and resist staining. PFCs are widely used in industrial applications as surfactants and emulsifiers and in consumer products such as food packaging, nonstick pan coatings, fire-fighting foams, paper and textile coatings, and personal care products (Calafat et al. 2007b; Renner 2001).

PFCs are extremely resistant to environmental and metabolic degradation and have been detected globally in the environment and wildlife (Lau et al. 2007). PFCs have been measured in the blood of occupationally exposed cohorts and in the general population. The source of PFCs in the general population is likely to be environmental exposure to individual PFCs or their precursor molecules; however, the specific source contributions are not well characterized. PFCs released during manufacturing processes or in wastes from the perfluoroalkyl industry are potential sources of exposure for the individuals employed in these industries as well as for the general population (3M Company 2003). Other potential sources of exposure include consumer products containing PFCs, contaminated drinking and surface waters, airborne PFCs, indoor air, and house dust (3M Company 2003; Boulanger et al. 2004; Emmett et al. 2006; Holzer et al. 2008; Martin et al. 2002; Saito et al. 2004; Shoeib et al. 2005; So et al. 2004; Steenland et al. 2009; Stock et al. 2004).

PFCs are absorbed through ingestion and to a lesser extent through inhalation. Once absorbed, PFCs are eliminated from the human body very slowly. Serum half-life estimates in an occupationally exposed cohort ranged from 5.4 years for perfluorooctane sulfonic acid (PFOS) to 8.5 years for perfluorohexane sulfonic acid (PFHxS) (Olsen et al. 2007). In a cohort exposed to drinking water contaminated by perfluorooctanoic acid (PFOA), the serum half-life for PFOA was estimated to be 2.3 years (Bartell et al. 2010). Although the primary producer of PFOS, the 3M Company, discontinued its use in 2002; and U.S. companies have implemented a voluntary emission reduction program for PFOA, > 98% of a 2003–2004 U.S. population sample had detectable serum levels of two perfluorinated carboxylates, PFOA and perfluorononanoic acid (PFNA), and two perfluorinated sulfonates, PFOS and PFHxS (Calafat et al. 2007b).

The ubiquitous presence and persistence of PFCs in the environment and the human body have led to efforts to understand the toxicologic hazards that may be associated with exposure. Early animal studies focused almost exclusively on exposure to PFOS and PFOA and found several potential effects, primarily related to hepatotoxicity, immunotoxicity, and reproductive and developmental toxicity (Lau et al. 2004, 2007). Although assessments are now including other PFCs and examining human populations, data are still limited.

Preliminary data suggest that PFCs may be potential developmental neurotoxicants. Using *in vitro* models, PFCs were shown to affect neuronal cell development in a variety of ways, including changes in cell differentiation (Slotkin et al. 2008). In rat models, *in utero* exposure to PFOS was linked to reduction in thyroid hormone (circulating thyroxin and triiodothyronine), which is known to regulate brain development (Lau et al. 2003; Luebker et al. 2005). However, in pups exposed to PFOS prenatally, reductions in thyroid hormone did not appear to disrupt learning and memory behavior in postnatal evaluations (Lau et al. 2003). Other developmental neurotoxic effects, manifested in changes in motor function and delayed learning, were observed in several animal studies (Fuentes et al. 2007a, 2007b; Johansson et al. 2008). Even at relatively low doses, Johansson et al. (2008) found developmental neurotoxic effects, including changes in spontaneous behavior and habituation ability, associated with PFOA and PFOS exposure in mice, which persisted into adulthood. Neonatal exposure to PFOS and PFOA has also been associated with changes in proteins (tau and synaptophysin) important in normal brain development (Johansson et al. 2009). To our knowledge, a single reported study assessed the human developmental neurotoxic effects of exposure to PFOA and PFOS. Using data for 1,400 pairs of pregnant women and their children randomly selected from the Danish National Birth Cohort, Fei et al. (2008) observed an association between maternal PFOS levels and delayed gross motor development in infancy (maternal report of the age at which a child could sit without support); however, maternal PFOS and PFOA levels were not significantly associated with delays in other developmental milestones, including those related to attention.

Attention deficit/hyperactivity disorder (ADHD) is one of the most common neurodevelopmental disorders in children, with an estimated prevalence between 7% and 16% in the United States (Faraone et al. 2003; Froehlich et al. 2007). Data suggest that the underlying prevalence of ADHD is increasing. Children diagnosed with ADHD are a heterogeneous population sharing common symptoms, including inattention, impulsivity, and, in some cases, hyperactivity, or a combination of symptoms. Although the mechanisms that lead to the development of ADHD remain unclear, genetic and environmental factors have been linked to ADHD. Environmental contaminants such as methylmercury and lead have been positively associated with ADHD in children (Braun et al. 2006; Cheuk and Wong 2006).

In the present analyses, we explored the association between ADHD and PFOS, PFOA, PFHxS, and PFNA using cross-sectional data from the National Health and Nutrition Examination Survey (NHANES) 1999–2000 and 2003–2004 cycles. To our knowledge, this is the first study examining the association between PFCs and ADHD.

## Methods

### Data source

NHANES is a nationally representative, cross-sectional sample of the noninstitutionalized U.S. civilian population. The survey combines in-home interviews and physical examinations in a mobile exam unit to collect data on demographics, socioeconomic status (SES), health conditions, and behavioral and environmental risk factors [Centers for Disease Controls and Prevention (CDC) 2008]. Details regarding interviews, examination procedures, and sample collection have been described previously (CDC 2009a, 2009b).

### ADHD and PFC exposure assessment

We used parental report of previous ADHD diagnosis as the primary dependent variable. Questionnaires were administered by trained personnel (CDC 2009a, 2009b). The children’s parents or guardians were asked if a doctor or health professional had ever told them that their child had attention deficit disorder. In NHANES, data on ADHD were collected in a target population of children 4–19 years of age.

To improve specificity, we also considered a second definition of ADHD used previously in an assessment of the effects of other environmental exposures on ADHD risk in the NHANES population (Braun et al. 2006). The second case definition included children with a parental report of a previous ADHD diagnosis and a parental report of their child taking medications approved for the treatment of ADHD within the preceding month (e.g., amphetamine aspartate, amphetamine sulfate, dextroamphetamine saccharate, dextroamphetamine sulfate, methylphenidate hydrochloride, or atomoxetine hydrochloride).

The National Center for Environmental Health analyzed serum PFC levels in a one-third sample of all individuals ≥ 12 years of age. Data were available during two nonconsecutive survey cycles, 1999–2000 and 2003–2004. Detailed analytic methods were described previously (Calafat et al. 2007a, 2007b). Briefly, serum samples were analyzed using automated solid-phase extraction coupled to reverse-phase high-performance liquid chromatography/tandem mass spectrometry. Any subject with a serum PFC concentration below the limit of detection (LOD) was assigned an exposure value of the LOD divided by the square root of 2 (Calafat et al. 2007b).

### Covariates

We investigated a number of covariates and potential confounders in the association between PFCs and ADHD. The demographic variables age, sex, and race/ethnicity were included as covariates based on their role in the NHANES selection procedure and previous research on their association with ADHD (Costello et al. 2003; Stevens et al. 2005). Additionally, we included NHANES sample cycle (1999–2000 or 2003–2004) as a covariate. SES was also considered a potential confounder. We used the poverty–income ratio (PIR), which relates the family income to the poverty threshold for each study year as a measure of SES. PIR values < 1.00 are considered living below the poverty level. Having a routine health care provider and health insurance coverage were also considered potential confounders (Stevens et al. 2005). In addition, we also investigated confounding by other environmental contaminants that have been previously associated with ADHD: lead and environmental tobacco smoke (ETS) indicated by report of living with someone who smokes cigarettes, cigars, or pipes inside the home and by serum cotinine, a metabolite of nicotine (Bellinger et al. 1994; Braun et al. 2006; Fergusson et al. 1988; Weitzman et al. 1992; Williams et al. 1998). We also considered confounding by variables related to conditions during the prenatal and early childhood periods: birth weight, admittance to a neonatal intensive care unit (NICU), maternal smoking during pregnancy, and preschool attendance (Botting et al. 1997; Braun et al. 2006; Mick et al. 2002a, 2002b; Milberger et al. 1996; National Institute of Child Health and Human Development 2003). NHANES collects different exposure, outcome, and confounder data depending on the age of the individual. Data on ADHD outcomes, confounder information, and PFC measurements were collected for children 12–15 years of age only; consequently, analyses were limited to this age range.

### Statistical analyses

We examined the shape of the relationship between the continuous measures of each individual PFC and parent-reported ADHD using a locally weighted regression smoother (LOESS) in S-Plus (version 8.0; Tibco Software, Inc., Palo Alto, CA). Smoothing allowed us to summarize ADHD odds as a function of PFC exposures without imposing a rigid form of dependence. We selected the optimal span size—the window from which data are drawn to estimate the odds of ADHD—by minimizing Akaike’s information criterion (Hastie and Tibshirani 1990). We visually inspected plots of the smoothed data to inform our decision on how to model exposure.

Logistic regression analyses were performed in SAS (version 9.1; SAS Institute, Inc., Cary, NC) using the Proc SURVEYLOGISTIC procedure, which accounts for stratification and clustering within primary sampling units used to select the NHANES sample. Rather than using NHANES sample weights, we adjusted all models for relevant covariates (age, sex, race/ethnicity, and sample cycle). This method is considered to be a good trade-off between efficiency and bias (Graubard and Korn 1999; Korn and Graubard 1991). Other potential covariates were included if they were strongly associated with ADHD in bivariate analyses (*p* < 0.10) or if they appreciably altered the association between PFC exposure and ADHD [odds ratio (OR) change > 10%]. We included continuous covariates in models as continuous predictors and also explored the use of categorization. A *p*-value of 0.05 was chosen to indicate the statistical significance of the association between each PFC and ADHD.

Our preliminary investigation of PFC data revealed several children with very high serum PFC levels, particularly PFHxS, PFOA, and PFNA. To ensure that individuals with extreme exposure values were not disproportionately influencing results, we conducted sensitivity analyses excluding observations greater than three times the 75th percentile for each compound (PFOS, *n* = 0; PFOA, *n* = 2; PFNA, *n* = 12; PFHxS, *n* = 26). Additionally, because the range of serum values varied considerably for the PFCs we assessed, we conducted additional analyses to provide ORs standardized to an increase in units equal to the interquartile range (IQR) for each PFC to provide comparable effect estimates.

We also investigated the impact of all four PFCs in the same model. Because PFC levels are correlated (in these data, Spearman correlations ranged from 0.18 between PFOS and PFNA to 0.74 between PFOS and PFOA), we also performed principal component analysis, a systematic method of reducing the number of correlated observed variables into a smaller number of principal components that account for most of the variance in the observed PFC measures (Burstyn 2004).

## Results

Of the 571 study participants 12–15 years of age with complete data, parents of 48 (8.4%) reported that their child had ADHD. Of those, 21 (3.6% of the study population) also reported using prescription medications approved for the treatment of ADHD within the last month. We included one child reported to be taking prescription medication for ADHD but not reported to have been diagnosed with ADHD in analyses as a noncase. Details regarding the total sample size are displayed in the Supplemental Material, Figure 1 (doi:10.1289/ehp.1001898).

Sex and maternal smoking during pregnancy were significantly associated with parental-report of ADHD in bivariate analyses (Table 1). Compared with non-Hispanic whites, Mexican Americans were less likely to report ADHD diagnosis [OR = 0.28; 95% confidence interval (CI), 0.11–0.72]. Associations between ETS and ADHD were similar whether ETS was indicated by categorical serum cotinine levels (data not shown) or report of living with someone who smokes cigarettes, cigars, or pipes inside the home. We controlled for ETS in models using report of living in a home with a smoker because data were missing less frequently. ETS was not associated with our stricter case definition of ADHD, having a reported diagnosis and taking prescription medication for the treatment of ADHD, which was similar to what Braun et al. (2006) observed. We also observed a positive association between lead and ADHD similar to that reported previously in a larger NHANES sample (Braun et al. 2006).

Table 2 displays the median serum level of each PFC in the study population. Nearly all study participants had detectable serum concentrations of all four PFCs included in our analyses (> 96% for all PFCs). Other PFCs were detected infrequently in this population. Table 3 displays the median serum PFC levels according to categorical covariates. Median serum concentrations were consistently higher in males than females and in children who attended preschool. Similarly, those who lived in a home with a smoker consistently had higher PFC levels. Table 4 displays correlations between continuous covariates and each PFC. With the exception of PFOA, which was weakly correlated with lead, we did not observe evidence of an association between PFCs and lead. Additionally, we observed a small but significant correlation between each PFC and the PIR.

The results of the smoothed analyses suggested that the association between PFC levels and ADHD may be approximately linear over most of the data range; accordingly, we included PFCs in logistic regression models as continuous predictors [see details in Supplemental Material, Figure 2 (doi:10.1289/ehp.1001898)]. We observed a significant (*p*-value < 0.05) dose–response relationship between PFOS exposure and parent-reported ADHD; the OR for each 1-μg/L increase in serum PFOS was 1.03 (95% CI, 1.01–1.05) (Table 5) after adjusting for confounding by NHANES sample cycle, age, race/ethnicity, sex, ETS, and maternal smoking during pregnancy. Crude estimates of the association between PFCs and ADHD were similar to adjusted estimates. The inclusion of other covariates, including the PIR, did not materially alter the association between PFCs and parent-reported ADHD (data not shown). PFOA and PFHxS levels were also positively associated with ADHD (PFOA: OR = 1.12; 95% CI, 1.01–1.23; PFHxS: OR = 1.06; 95% CI, 1.02–1.11). The odds of parent-reported ADHD also increased with PFNA concentrations, although not significantly (OR = 1.32; 95% CI, 0.86–2.02). Results were similar when we used the stricter case definition of ADHD that required both parental report and medication use (Table 5). The same covariates were evaluated for each case definition.

Because the range of serum values varied considerably for the PFCs we assessed, we also calculated ORs standardized to an increase in units equal to the IQR for each PFC. An increase in serum PFOS levels equal to the IQR was associated with 1.60 times the odds of ADHD (95% CI, 1.10–2.31; IQR = 15.9 μg/L). For PFOA, the odds of ADHD increased 1.35 times for an increase equal to the IQR (95% CI, 1.04–1.77; IQR = 2.7 μg/L). A 2.9-μg/L increase in serum PFHxS increased the odds of ADHD 1.19 times (95% CI, 1.05–1.34). The IQR effect for PFNA increased ADHD odds 1.15 times (95% CI, 0.93–1.42; IQR = 0.5 μg/L).

Several children had very high serum PFC levels, particularly PFHxS, PFOA, and PFNA. Estimated ORs were slightly higher than those reported in Table 5 when observations with PFC concentrations more than three times the value of the 75th percentile were excluded (data not shown).

Principal component analyses indicated that PFOS, PFOA, and PFHxS all loaded onto a single component that accounted for 58% of the total variability in all four PFC measures. A second component, which primarily represented PFNA exposure, accounted for an additional 22% of the total variability. We repeated multiple logistic regression analyses including both PFNA and the principal component that represented the weighted combination of PFOS, PFOA, and PFHxS. A positive association with ADHD remained for the combined PFC variable and for PFNA (data not shown; PFOS, PFOA, and PFHxS component *p*-value = 0.02; PFNA *p*-value = 0.72).

## Discussion

We observed a positive dose–response relationship between parent-reported ADHD and serum PFOS, PFOA, and PFHxS concentrations modeled as continuous predictors. Including PFC levels modeled as categorical predictors of ADHD produced similar results. The estimated effect of exposure on the population level was similar across these PFCs based on IQR analyses, indicating the importance of extending neurotoxicologic research to PFCs other than PFOS and PFOA, which have historically been the focus of research.

Principal component analyses support evidence of a positive association between ADHD and serum PFCs. Controlling for PFNA levels and other covariates, we observed a significant positive association with the principal component representing PFOS, PFOA, and PFHxS, suggesting that there may be different sources of exposure for PFNA and the other PFCs that we assessed. This is an area for future research.

Our results are consistent with animal data that suggest neurotoxic effects of PFC exposures (Fuentes et al. 2007a, 2007b; Johansson et al. 2008). To our knowledge, the report by Fei et al. (2008) is the only other published study of neurodevelopmental outcomes and PFC exposures. Except for gross motor ability, Fei et al. (2008) did not observe statistically significant differences in maternal reports of developmental milestones in infancy related to PFOS or PFOA exposure. The age of individuals in the study populations may explain the difference between our results and those reported by Fei et al. (2008). Because of data availability, we were able to assess only children 12–15 years of age.

Our analyses have a number of limitations. NHANES data are collected cross-sectionally, making it difficult to infer a causal relationship between PFC levels and ADHD. These data do not allow us to speculate on potential development periods of susceptibility to exposure. Although PFCs have a relatively long half-life, there is a lack of information addressing whether current PFC levels are an appropriate surrogate for past levels in the general population (Bartell et al. 2010; Olsen et al. 2007). If the prenatal period and early childhood, when the developing brain may be more susceptible, are the critical periods for insult from PFC exposure, then random misclassification error resulting from the use of current PFC levels as proxy measures of etiologically relevant exposures may have biased our results toward the null.

These analyses are also potentially limited by our reliance on parental report of ADHD. Previous research indicates parental reports of ADHD are highly reliable (Faraone et al. 1995). Examining the combined outcome of reported ADHD and medication use, we improved the specificity of the outcome and increased the likelihood that children included as ADHD cases had been evaluated and treated by health care professionals. Although we identified a much smaller number of cases using the stricter definition (*n* = 21), and consequently reduced the precision, the point estimates were similar regardless of which case definition was used. Similarly, although we included a large number of terms in models for a relatively small number of outcomes, crude estimates of the association between PFCs and ADHD were similar to adjusted estimates. Analyses of the NHANES population conducted previously have shown that parental report of ADHD was related to other environmental exposures (e.g., lead and maternal smoking during pregnancy) (Braun et al. 2006).

Detailed questionnaire and laboratory measurement data were available for a number of potential covariates in the NHANES data set, and we able to evaluate the potential for confounding by conditions during the prenatal and early childhood periods and other environmental exposures, such as lead and SES, related to ADHD. Data for potential confounders were generally very complete. However, there may still be residual confounding in the association between PFCs and ADHD. We were unable to assess several potential confounders because they were either not publically available (i.e., maternal alcohol consumption during pregnancy) or not collected in the NHANES data set (i.e., genetic predisposition for ADHD).

Despite these limitations, our analyses have a number of strengths including the use of a representative sample of the U.S. population. Additionally, we were able to consider the association between PFC levels and ADHD odds over a wide range of exposures. We were also able to estimate associations of PFNA and PFHxS with ADHD; to our knowledge, this is the first assessment of the potential developmental neurotoxicity of these PFCs in children.

## Conclusions

To our knowledge, these analyses are the first to assess the association between PFC levels and ADHD. As a whole, our results are consistent with increased ADHD in children with higher serum levels of PFCs. Given the extremely prevalent exposure to PFCs, further investigation into the impact of PFC exposure on ADHD and other neurodevelopmental endpoints is warranted.

## Figures and Tables

**Table 1:** Selected population characteristics and bivariate analyses of parental-reported ADHD and covariates.

Variable	Cases	Noncases	OR (95% CI)
Age [mean (years)]	13.4	13.4	0.93 (0.73–1.18)
Sex (*n*)			
Male	41	255	4.50 (2.17–9.37)
Female	10	280	Reference
Race/ethnicity (*n*)			
Mexican American	9	206	0.28 (0.11–0.72)
Other Hispanic	1	25	0.26 (0.03–2.11)
Non-Hispanic white	18	116	Reference
Non-Hispanic black	20	164	0.79 (0.35–1.76)
Other, including multiracial	3	24	0.81 (0.28–2.33)
Birth weight (*n*)^a^,^b^			
≤ 5.5 lb (2,500 g)	4	26	1.57 (0.62–4.02)
> 5.5 lb (2,500 g)	47	481	Reference
Maternal smoking during pregnancy (*n*)^a^			
Yes	13	80	2.08 (1.04–4.17)
No	35	448	Reference
Preschool attendance (*n*)^a^			
Yes	37	341	1.50 (0.84–2.68)
No	14	193	Reference
NICU admittance (*n*)			
Yes	8	60	1.45 (0.83–2.53)
No	43	467	Reference
ETS (*n*)^c^			
Yes	22	117	2.68 (1.58–4.53)
No	29	413	Reference
Lead [mean (μg/dL)]^a^	1.5	1.3	1.09 (0.90–1.34)
PIR (mean)^a^	1.8	1.9	1.08 (0.94–1.23)
Access to health care (*n*)^d^			
Yes	49	495	1.98 (0.71–5.49)
No	2	40	Reference
Health insurance coverage (*n*)^a^			
Yes	46	427	2.15 (0.83–5.57)
No	5	100	Reference
NHANES sample wave (*n*)			
1999–2000	20	258	Reference
2003–2004	31	277	1.44 (0.84–2.48)

a Missing data: low birth weight, *n* = 28; maternal smoking during pregnancy, *n* = 10; preschool attendance, *n* = 1; ETS, *n* = 5; lead, *n* = 1; PIR, *n* = 35; health insurance coverage, *n* = 8.

b Low birth weight defined as ≤ 5.5 lb or 2,500 g.

c Report of living in a home with someone who smokes cigarettes, cigars, or pipes inside.

d Parents reported that the child had one or more places to go when they were sick or need advice about health.

**Table 2:** Distribution of PFC levels in the study population (μg/L).

Variable	Median	Range	IQR
PFOS	22.6	2.1–87.2	15.9
PFOA	4.4	0.4–21.7	2.7
PFHxS	2.2	ND to 64.1	2.9
PFNA	0.6	ND to 5.9	0.5

ND, nondetectable. The LOD in 1999–2000 serum samples was 0.2 μg/L for PFOS, 0.1 μg/L for PFOA, 0.1 μg/L for PFHxS, and 0.1 μg/L for PFNA; in 2003–2004 serum samples, it was 0.4 μg/L for PFOS, 0.1 μg/L for PFOA, 0.3 μg/L for PFHxS, and 0.1 μg/L for PFNA (Calafat et al. 2007b).

**Table 3:** Median serum PFC concentrations (μg/L) by categorical covariates.

Variable	*n*	PFOS	PFOA	PFHxS	PFNA
Sex					
Male	296	23.7	4.8	2.5	0.7
Female	290	21.9	4.0	2.0	0.6
Race/ethnicity					
Mexican American	215	20.0	4.1	1.7	0.4
Other Hispanic	26	18.8	4.4	1.3	0.5
Non-Hispanic white	134	26.3	4.6	3.3	0.7
Non-Hispanic black	184	24.1	4.6	2.4	0.8
Other, including multiracial	27	24.6	4.1	2.7	0.5
Birth weight^a^					
≤ 5.5 lb (2,500 g)	30	22.0	3.8	2.3	0.7
> 5.5 lb (2,500 g)	528	22.7	4.4	2.2	0.6
Maternal smoking during pregnancy					
Yes	93	22.8	4.4	2.6	0.7
No	483	22.5	4.1	2.1	0.6
Preschool attendance^a^					
Yes	378	23.5	4.5	2.4	0.7
No	207	21.1	4.1	1.8	0.5
NICU admittance					
Yes	68	22.4	4.5	2.5	0.6
No	510	22.6	4.4	2.2	0.6
ETS^a^					
Yes	139	24.7	4.5	2.3	0.7
No	442	22.2	4.3	2.1	0.6
Access to health care					
Yes	544	23.0	4.4	2.3	0.6
No	42	18.4	3.5	1.4	0.4
Health insurance coverage^a^					
Yes	473	23.4	4.4	2.3	0.6
No	105	18.8	3.9	1.6	0.4
NHANES sample wave					
1999–2000	278	28.2	5.3	2.2	0.4
2003–2004	308	18.2	3.8	2.2	0.8

a Missing data: low birth weight, *n* = 28; maternal smoking during pregnancy, *n* = 10; preschool attendance, *n* = 1; ETS, *n* = 5; health insurance coverage, *n* = 8.

**Table 4:** Spearman correlations between continuous covariates and PFCs (*p*-value).

Variable	PFOS	PFOA	PFHxS	PFNA
Age (years)	0.078 (0.059)	−0.051 (0.220)	−0.052 (0.208)	0.008 (0.852)
Lead^a^	0.062 (0.133)	0.121 (0.003)	0.033 (0.428)	0.030 (0.464)
PIR^a^	0.233 (< 0.001)	0.172 (< 0.001)	0.178 (< 0.001)	0.169 (< 0.001)

a Missing data: lead, *n* = 1; PIR, *n* = 35.

**Table 5:** ORs (95% CIs) for 1-μg/L increase in serum level (*n* = 571).

	Parental report of ADHD		With prescription medication use (adjusted)^a^
	Crude	Adjusted^a^	
PFOS	1.03 (1.01–1.05)	1.03 (1.01–1.05)	1.05 (1.02–1.08)
PFOA	1.17 (1.07–1.30)	1.12 (1.01–1.23)	1.19 (0.95–1.49)
PFHxS	1.07 (1.01–1.12)	1.06 (1.02–1.11)	1.07 (1.03–1.11)
PFNA	1.76 (1.39–2.23)	1.32 (0.86–2.02)	1.57 (0.67–3.64)

a Adjusted for NHANES sample cycle, age, sex, race, ETS, and maternal smoking during pregnancy.

## References

[b1-ehp-118-1762] 3M Company (2003). Environmental and Health Assessment of Perfluorooctane Sulfonic Acid and Its Salts. AR 226-1486.

[b2-ehp-118-1762] Bartell SM, Calafat AM, Lyu C, Kato K, Ryan B, Steenland K (2010). Rate of decline in serum PFOA concentrations after granular activated carbon filtration at two public water systems in Ohio and West Virginia. Environ Health Perspect.

[b3-ehp-118-1762] Bellinger D, Leviton A, Allred E, Rabinowitz M (1994). Pre- and postnatal lead exposure and behavior problems in school-aged children. Environ Res.

[b4-ehp-118-1762] Botting N, Powls A, Cooke RW, Marlow N (1997). Attention deficit hyperactivity disorders and other psychiatric outcomes in very low birthweight children at 12 years. J Child Psychol Psychiatry.

[b5-ehp-118-1762] Boulanger B, Vargo J, Schnoor JL, Hornbuckle KC (2004). Detection of perfluorooctane surfactants in Great Lakes water. Environ Sci Technol.

[b6-ehp-118-1762] Braun JM, Kahn RS, Froehlich T, Auinger P, Lanphear BP (2006). Exposures to environmental toxicants and attention deficit hyperactivity disorder in U.S. children. Environ Health Perspect.

[b7-ehp-118-1762] Burstyn I (2004). Principal component analysis is a powerful instrument in occupational hygiene inquiries. Ann Occup Hyg.

[b8-ehp-118-1762] Calafat AM, Kuklenyik Z, Reidy JA, Caudill SP, Tully JS, Needham LL (2007a). Serum concentrations of 11 polyfluoroalkyl compounds in the U.S. population: data from the National Health and Nutrition Examination Survey (NHANES). Environ Sci Technol.

[b9-ehp-118-1762] Calafat AM, Wong LY, Kuklenyik Z, Reidy JA, Needham LL (2007b). Polyfluoroalkyl chemicals in the U.S. population: data from the National Health and Nutrition Examination Survey (NHANES) 2003–2004 and comparisons with NHANES 1999–2000. Environ Health Perspect.

[b10-ehp-118-1762] CDC (Centers for Disease Control and Prevention) (2008). National Health and Nutrition Examination Survey data.

[b11-ehp-118-1762] CDC (Centers for Disease Control and Prevention) (2009a). Survey Questionnaires, Examination Components and Laboratory Components 2003–2004.

[b12-ehp-118-1762] CDC (Centers for Disease Control and Prevention) (2009b). Survey Questionnaires, Examination Components and Laboratory Components 1999–2000.

[b13-ehp-118-1762] Cheuk DK, Wong V (2006). Attention-deficit hyperactivity disorder and blood mercury level: a case-control study in Chinese children. Neuropediatrics.

[b14-ehp-118-1762] Costello EJ, Mustillo S, Erkanli A, Keeler G, Angold A (2003). Prevalence and development of psychiatric disorders in childhood and adolescence. Arch Gen Psychiatry.

[b15-ehp-118-1762] Emmett EA, Shofer FS, Zhang H, Freeman D, Desai C, Shaw LM (2006). Community exposure to perfluorooctanoate: relationships between serum concentrations and exposure sources. J Occup Environ Med.

[b16-ehp-118-1762] Faraone SV, Biederman J, Milberger S (1995). How reliable are maternal reports of their children’s psychopathology? One-year recall of psychiatric diagnoses of ADHD children. J Am Acad Child Adolesc Psychiatry.

[b17-ehp-118-1762] Faraone SV, Sergeant J, Gillberg C, Biederman J (2003). The worldwide prevalence of ADHD: is it an American condition?. World Psychiatry.

[b18-ehp-118-1762] Fei C, McLaughlin JK, Lipworth L, Olsen J (2008). Prenatal exposure to perfluorooctanoate (PFOA) and perfluorooctanesulfonate (PFOS) and maternally reported developmental milestones in infancy. Environ Health Perspect.

[b19-ehp-118-1762] Fergusson DM, Fergusson JE, Horwood LJ, Kinzett NG (1988). A longitudinal study of dentine lead levels, intelligence, school performance and behaviour. Part III. Dentine lead levels and attention/activity. J Child Psychol Psychiatry.

[b20-ehp-118-1762] Froehlich TE, Lanphear BP, Epstein JN, Barbaresi WJ, Katusic SK, Kahn RS (2007). Prevalence, recognition, and treatment of attention-deficit/hyperactivity disorder in a national sample of US children. Arch Pediatr Adolesc Med.

[b21-ehp-118-1762] Fuentes S, Colomina MT, Vicens P, Domingo JL (2007a). Influence of maternal restraint stress on the long-lasting effects induced by prenatal exposure to perfluorooctane sulfonate (PFOS) in mice. Toxicol Lett.

[b22-ehp-118-1762] Fuentes S, Vicens P, Colomina MT, Domingo JL (2007b). Behavioral effects in adult mice exposed to perfluorooctane sulfonate (PFOS). Toxicology.

[b23-ehp-118-1762] Graubard BI, Korn EL (1999). Analyzing health surveys for cancer-related objectives. J Natl Cancer Inst.

[b24-ehp-118-1762] Hastie T, Tibshirani R (1990). Generalized Additive Models.

[b25-ehp-118-1762] Holzer J, Midasch O, Rauchfuss K, Kraft M, Reupert R, Angerer J (2008). Biomonitoring of perfluorinated compounds in children and adults exposed to perfluorooctanoate-contaminated drinking water. Environ Health Perspect.

[b26-ehp-118-1762] Johansson N, Eriksson P, Viberg H (2009). Neonatal exposure to PFOS and PFOA in mice results in changes in proteins which are important for neuronal growth and synaptogenesis in the developing brain. Toxicol Sci.

[b27-ehp-118-1762] Johansson N, Fredriksson A, Eriksson P (2008). Neonatal exposure to perfluorooctane sulfonate (PFOS) and perfluorooctanoic acid (PFOA) causes neurobehavioural defects in adult mice. Neurotoxicology.

[b28-ehp-118-1762] Korn EL, Graubard BI (1991). Epidemiologic studies utilizing surveys: accounting for the sampling design. Am J Public Health.

[b29-ehp-118-1762] Lau C, Anitole K, Hodes C, Lai D, Pfahles-Hutchens A, Seed J (2007). Perfluoroalkyl acids: a review of monitoring and toxicological findings. Toxicol Sci.

[b30-ehp-118-1762] Lau C, Butenhoff JL, Rogers JM (2004). The developmental toxicity of perfluoroalkyl acids and their derivatives. Toxicol Appl Pharmacol.

[b31-ehp-118-1762] Lau C, Thibodeaux JR, Hanson RG, Rogers JM, Grey BE, Stanton ME (2003). Exposure to perfluorooctane sulfonate during pregnancy in rat and mouse. II: Postnatal evaluation. Toxicol Sci.

[b32-ehp-118-1762] Luebker DJ, York RG, Hansen KJ, Moore JA, Butenhoff JL (2005). Neonatal mortality from *in utero* exposure to perfluorooctanesulfonate (PFOS) in Sprague-Dawley rats: dose-response, and biochemical and pharamacokinetic parameters. Toxicology.

[b33-ehp-118-1762] Martin JW, Muir DC, Moody CA, Ellis DA, Kwan WC, Solomon KR (2002). Collection of airborne fluorinated organics and analysis by gas chromatography/chemical ionization mass spectrometry. Anal Chem.

[b34-ehp-118-1762] Mick E, Biederman J, Faraone SV, Sayer J, Kleinman S (2002a). Case-control study of attention-deficit hyperactivity disorder and maternal smoking, alcohol use, and drug use during pregnancy. J Am Acad Child Adolesc Psychiatry.

[b35-ehp-118-1762] Mick E, Biederman J, Prince J, Fischer MJ, Faraone SV (2002b). Impact of low birth weight on attention-deficit hyperactivity disorder. J Dev Behav Pediatr.

[b36-ehp-118-1762] Milberger S, Biederman J, Faraone SV, Chen L, Jones J (1996). Is maternal smoking during pregnancy a risk factor for attention deficit hyperactivity disorder in children?. Am J Psychiatry.

[b37-ehp-118-1762] National Institute of Child Health and Human Development (2003). Does amount of time spent in child care predict socioemotional adjustment during the transition to kindergarten?. Child Dev.

[b38-ehp-118-1762] Olsen GW, Burris JM, Ehresman DJ, Froehlich JW, Seacat AM, Butenhoff JL (2007). Half-life of serum elimination of perfluorooctanesulfonate, perfluorohexanesulfonate, and perfluorooctanoate in retired fluorochemical production workers. Environ Health Perspect.

[b39-ehp-118-1762] Renner R (2001). Growing concern over perfluorinated chemicals. Environ Sci Technol.

[b40-ehp-118-1762] Saito N, Harada K, Inoue K, Sasaki K, Yoshinaga T, Koizumi A (2004). Perfluorooctanoate and perfluorooctane sulfonate concentrations in surface water in Japan. J Occup Health.

[b41-ehp-118-1762] Shoeib M, Harner T, Wilford BH, Jones KC, Zhu J (2005). Perfluorinated sulfonamides in indoor and outdoor air and indoor dust: occurrence, partitioning, and human exposure. Environ Sci Technol.

[b42-ehp-118-1762] Slotkin TA, MacKillop EA, Melnick RL, Thayer KA, Seidler FJ (2008). Developmental neurotoxicity of perfluorinated chemicals modeled *in vitro*. Environ Health Perspect.

[b43-ehp-118-1762] So MK, Taniyasu S, Yamashita N, Giesy JP, Zheng J, Fang Z (2004). Perfluorinated compounds in coastal waters of Hong Kong, South China, and Korea. Environ Sci Technol.

[b44-ehp-118-1762] Steenland K, Jin C, MacNeil J, Lally C, Ducatman A, Vieira V (2009). Predictors of PFOA levels in a community surrounding a chemical plant. Environ Health Perspect.

[b45-ehp-118-1762] Stevens J, Harman JS, Kelleher KJ (2005). Race/ethnicity and insurance status as factors associated with ADHD treatment patterns. J Child Adolesc Psychopharmacol.

[b46-ehp-118-1762] Stock NL, Lau FK, Ellis DA, Martin JW, Muir DC, Mabury SA (2004). Polyfluorinated telomer alcohols and sulfonamides in the North American troposphere. Environ Sci Technol.

[b47-ehp-118-1762] Weitzman M, Gortmaker S, Sobol A (1992). Maternal smoking and behavior problems of children. Pediatrics.

[b48-ehp-118-1762] Williams GM, O’Callaghan M, Najman JM, Bor W, Andersen MJ, Richards D (1998). Maternal cigarette smoking and child psychiatric morbidity: a longitudinal study. Pediatrics.

